# m^7^G-quant-seq: Quantitative Detection of RNA Internal *N*^7^-Methylguanosine

**DOI:** 10.1021/acschembio.2c00792

**Published:** 2022-11-18

**Authors:** Li-Sheng Zhang, Cheng-Wei Ju, Chang Liu, Jiangbo Wei, Qing Dai, Li Chen, Chang Ye, Chuan He

**Affiliations:** † Department of Chemistry, The University of Chicago, Chicago, Illinois 60637, United States; ‡ Division of Life Science, Department of Chemistry, The Hong Kong University of Science and Technology, Clear Water Bay, Kowloon, Hong Kong SAR 999077, China; § Pritzker School of Molecular Engineering, The University of Chicago, Chicago, Illinois 60637, United States; ∥ Howard Hughes Medical Institute, 4000 Jones Bridge Road, Chevy Chase, Maryland 20815, United States

## Abstract

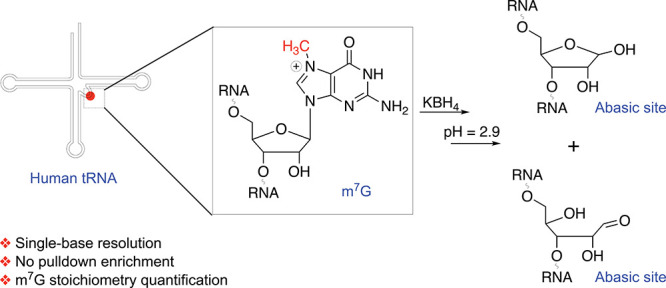

Methods for the precise detection and quantification of RNA modifications are critical to uncover functional roles of diverse RNA modifications. The internal m^7^G modification in mammalian cytoplasmic tRNAs is known to affect tRNA function and impact embryonic stem cell self-renewal, tumorigenesis, cancer progression, and other cellular processes. Here, we introduce m^7^G-quant-seq, a quantitative method that accurately detects internal m^7^G sites in human cytoplasmic tRNAs at single-base resolution. The efficient chemical reduction and mild depurination can almost completely convert internal m^7^G sites into RNA abasic sites (AP sites). We demonstrate that RNA abasic sites induce a mixed variation pattern during reverse transcription, including G → A or C or T mutations as well as deletions. We calculated the total variation ratio to quantify the m^7^G modification fraction at each methylated site. The calibration curves of all relevant motif contexts allow us to more quantitatively determine the m^7^G methylation level. We detected internal m^7^G sites in 22 human cytoplasmic tRNAs from HeLa and HEK293T cells and successfully estimated the corresponding m^7^G methylation stoichiometry. m^7^G-quant-seq could be applied to monitor the tRNA m^7^G methylation level change in diverse biological processes.

## Introduction

Transfer RNA (tRNA), as one of the abundant noncoding RNAs, is subject to numerous post-transcriptional modifications that regulate tRNA biogenesis, structural folding, stability, and function.^1,2^ Dysregulation of tRNA modification is linked to neurological disorders, mitochondrial disease, type 2 diabetes, and cancer.^3,4^ Internal *N*^7^-methylguanosine (m^7^G) at the nucleotide position 46 of tRNA (m^7^G46) is one of the most prevalent tRNA modifications, which is responsible for the tertiary base-pairing with C13-G22 that stabilizes the tRNA structure.^5,6^

A heterodimer of METTL1/WDR4 was identified as a “writer” machinery that installs a tRNA m^7^G modification in eukaryotes.^7−10^ The mutation in human WDR4 impairs tRNA m^7^G methylation and causes microcephalic primordial dwarfism.^11^ tRNA m^7^G methylation also affects embryonic stem cell self-renewal and differentiation.^12,13^ METTL1 is frequently overexpressed in human cancers and is associated with poor patient survival in some cancers; METTL1 depletion inhibits oncogenicity and tumor growth in many cancer types.^14^ For example, the METTL1-mediated tRNA m^7^G was shown to promote cancer progression in hepatocellular carcinoma and intrahepatic cholangiocarcinoma^15,16^ and enhance esophageal squamous cell carcinoma tumorigenesis.^17^

To study internal m^7^G profiles in various human tRNAs by high-throughput sequencing, chemical-assisted approaches have been proposed to detect tRNA m^7^G modification at base resolution. In 2018, Marchand et al. reported alkaline hydrolysis and aniline cleavage sequencing (AlkAniline-Seq) to measure internal m^7^G and m^3^C in one pot, based on truncation signatures generated during reverse transcription (RT).^18^ In the same year, using a similar principle, Lin et al. developed tRNA reduction and cleavage sequencing (TRAC-Seq) to globally map tRNA m^7^G at single-nucleotide resolution, via NaBH_4_-induced reduction and aniline-promoted RNA cleavage.^12,19^ Different from such chemical cleavage-mediated detection, in 2019 our lab reported m^7^G-seq by the application of chemical reduction and depurination, which selectively converts internal m^7^G sites into biotinylated abasic sites (AP sites).^20,21^ Using human immunodeficiency virus (HIV) reverse transcriptase, m^7^G-seq successfully detects internal m^7^G sites on human tRNA as RT misincorporation signatures, at 20–60% mutation ratios after biotin pulldown.^20^ The method also revealed notable m^7^G methylation on mRNA from several cancer cell lines. In a back-to-back report, Pandolfini et al. employed a similar principle and discovered internal m^7^G methylation within human *let-7* miRNA, which is installed by METTL1.^22^ In the same year, Enroth et al. published m^7^G-MaP-seq, which directly converts m^7^G-modified positions into abasic sites by one-step NaBH_4_ reduction and read out as cDNA mutations.^23^ However, the <8% misincorporation levels at tRNA m^7^G sites revealed in m^7^G-MaP-seq are a bit too low to monitor methylation level change at all m^7^G sites in human tRNA.

Despite these recent advances in m^7^G sequencing method development, we still lack a quantitative method that maps m^7^G sites with stoichiometric information and high sensitivity, mostly because of incomplete reduction, cleavage, or depurination at internal m^7^G sites. In addition, the presence of multiple heavily modified bases on tRNA and its extensive secondary structure also challenge the direct sequencing of tRNA m^7^G.^24^ Our recent work on RT-misincorporation-based m^1^A-quant-seq^25^ and m^6^A-SAC-seq^26^ to achieve more accurate sequencing of m^1^A and m^6^A methylations in mRNA inspired us to continue optimizing the RT-misincorporation-based m^7^G-seq. Also, the recent successes in quantification of m^1^A, m^3^C, m^1^G, and m^2^_2_G methylations in cytoplasmic and mitochondrial tRNAs with excellent read-through across the methylated regions, such as DAMM-seq,^27^ PANDORA-seq,^28^ and AQRNA-seq,^29^ provided strategies to overcome challenges in tRNA sequencing. Here, we introduce m^7^G-quant-seq, a method that detects abasic sites derived from internal m^7^G modifications on human tRNA at single-base resolution with stoichiometry information.

## Results

### m^7^G-quant-seq Detects Human 18S rRNA m^7^G1639 in High Variation Ratios

**Figure 1:**
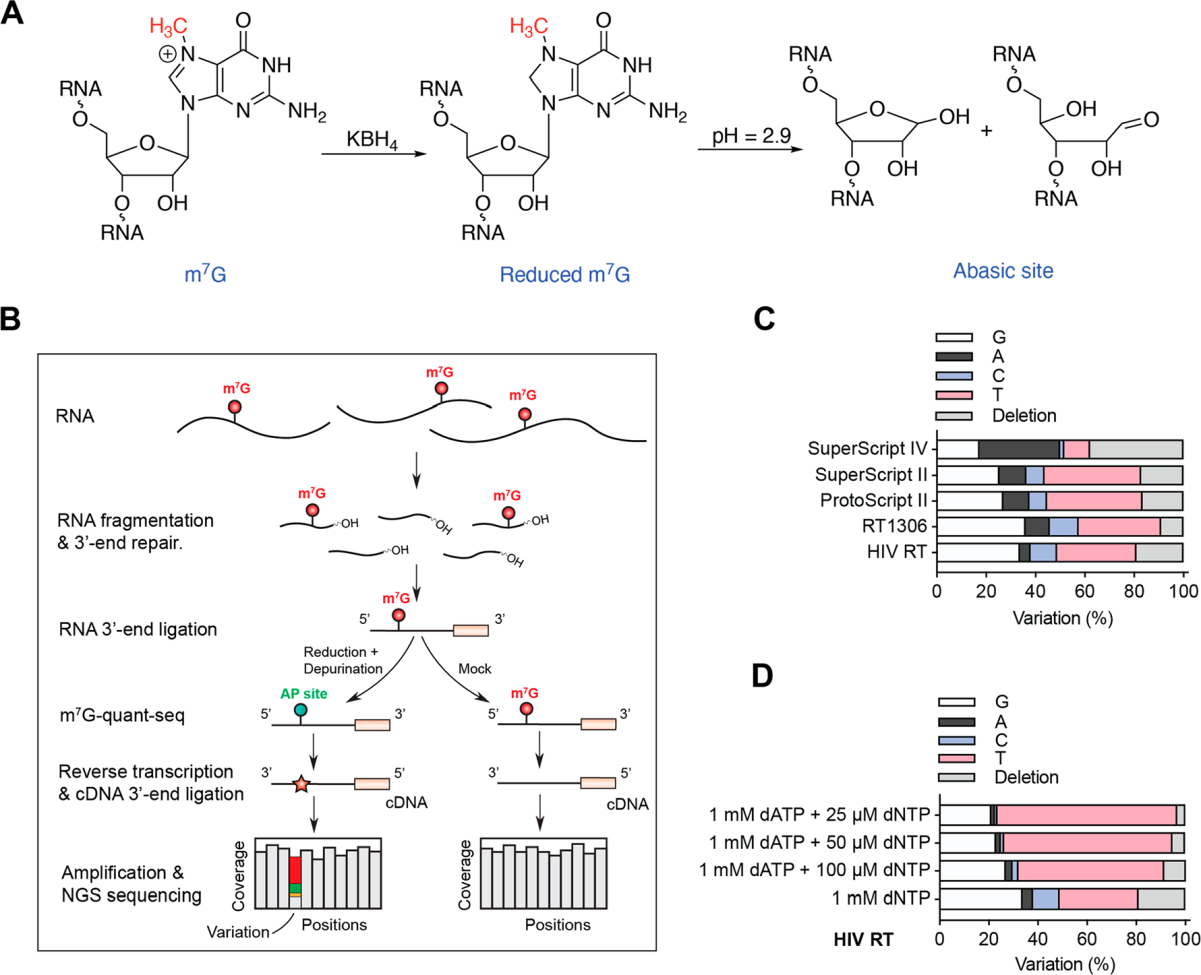
**Development of m**^**7**^**G-quant-seq**. (A) Chemical structures of reduced m^7^G and RNA abasic site, generated under m^7^G-quant-seq treatment. KBH_4_-mediated reduction at room temperature for 4 h, followed by a mild depurination (pH = 2.9) at 45 °C for 4 h. (B) A flowchart of library construction in m^7^G-quant-seq, revealing m^7^G methylation fraction by the sum of variation signatures. (C) The variation signatures at the AP site generated from HeLa 18S rRNA m^7^G1639, under m^7^G-quant-seq treatment and with different RTs. (D) The variation signatures at the AP site generated from HeLa 18S rRNA m^7^G1639, under m^7^G-quant-seq treatment with wild-type HIV RT and adjusted dNTP/dATP ratios.

Although m^7^G-seq can estimate the modification fraction of internal m^7^G sites on human tRNAs in the absence of biotin pulldown, the ∼10–20% misincorporation ratios are not high enough for either accurate quantification of m^7^G stoichiometry or sensitive measurement of m^7^G methylation fraction change.^20,21^ We attributed this to incomplete conversion of internal m^7^G sites into RNA abasic sites (AP sites), under the m^7^G-seq chemical treatment conditions. To elevate the misincorporation ratios at an internal m^7^G site, we optimized and performed two improvements (Figure 1A), including high-efficiency reduction and depurination. First, we utilized KBH_4_-mediated reduction with high BH_4_^–^ concentration (∼800 mM) at room temperature for 4 h to completely convert m^7^G into its reduced form. We synthesized a single-stranded RNA (ssRNA) oligo probe containing a single internal m^7^G site, which was designed to be free of secondary structures nearby. Both m^7^G-quant-seq KBH_4_ treatment and m^7^G-seq NaBH_4_ treatment^20,21^ gave a ∼99% reduction efficiency at the internal m^7^G site within the synthetic RNA probe (Figure S1A). We then tested the fragmented HeLa total RNA, which may contain some secondary structures around the internal m^7^G site. While m^7^G-seq NaBH_4_ treatment^20,21^ led to ∼77% reduction of m^7^G, m^7^G-quant-seq KBH_4_ treatment converted ∼97% m^7^G into reduced m^7^G (Figures 1A and S1B). Taken together, m^7^G-quant-seq KBH_4_ treatment showed a much higher reduction efficiency. Second, we employed a mild depurination condition at pH = 2.9 (100 mM NaOAc/AcOH buffer) with heating at 45 °C for 4 h to generate a stable RNA abasic site at the reduced m^7^G site, with two resonant structures that could further induce misincorporation or deletion signatures during reverse transcription (Figure 1A).

Based on the new chemical treatment we measured variation signals generated at AP site during reverse transcription to comprehensively detect and quantify internal m^7^G sites in biological samples (Figure 1B).

To validate the improvement, we applied m^7^G-quant-seq to HeLa total RNA with the wild-type HIV RT and captured a mixed variation pattern at the AP site generated from 18S rRNA m^7^G1639, which includes 4.3% G → A mutation, 10.8% G → C mutation, 32.1% G → T mutation, and 19.1% deletion (∼66% variation in total, Figure 1C). This is a dramatic improvement compared with the ∼30% mutation ratio (without biotin pulldown enrichment) previously observed in m^7^G-seq.^20^ We then screened three commercially available RTs plus one engineered RT and obtained 72.3%, 74.6%, 82.6%, and 64.0% variation ratios for ProtoScript II RT, SuperScript II RT, SuperScript IV RT, and RT1306,^25^ respectively (Figure 1C). Overall, at this 18S rRNA m^7^G site (in AG(m^7^G)AA motif), G → T mutation is the major variation type for HIV RT, ProtoScript II RT, SuperScript II RT, and RT1306, while SuperScript IV RT shows G → A mutation plus deletion as its main variation signature (Figure 1C). The variation signatures are nearly the same between SuperScript II RT and ProtoScript II RT (Figure 1C).

Note that, in m^7^G-seq,^20,21^ we only observed a ∼30% misincorporation ratio at m^7^G1639 (within an AG(m7G)AA motif) in wild-type HeLa 18S rRNA. Although m^7^G-quant-seq revealed an ∼66% total mutation and deletion ratio at the 18S m^7^G1639 site, which corresponds to at least 85% m^7^G methylation fraction in wild-type HeLa cells (Figure S1C), we detected a ∼79% total mutation and deletion ratio, corresponding to ∼99% m^7^G methylation fraction, at the 18S m^7^G_1639_ site in total RNA from HeLa shControl cells. This measured total mutation and deletion rate is close to the ∼83% variation ratio obtained from the synthetic RNA oligo with the AG(abasic site)AA motif (Figure S1C). These results suggested the almost complete conversion of the internal m^7^G site into an RNA abasic site after two steps of chemical treatment in m^7^G-quant-seq.

Because G → T mutation is the major variation type for most RTs, we next tested different deoxyribonucleoside triphosphate (dNTP)/deoxyadenosine triphosphate (dATP) ratios to further elevate the variation rates and to tune the variation pattern at the AP site generated from 18S m^7^G1639. Starting with HIV RT, we observed obviously elevated G → T mutation ratios (as 64.0%, 68.6%, and 73.3%) and globally increased total variation ratios (as 73.2%, 76.9%, and 79.2%) when adjusting dNTP/dATP ratios to 100, 50, and 25 μM/1 mM, respectively (Figure 1D). These results reveal that elevated dATP/dNTP ratios can largely improve the overall variation rate at internal m^7^G sites and can enhance the variation signature of the G → T mutation. We observed similar effects when testing RT1306, ProtoScript II, and SuperScript II (Figure S1D–F). Because SuperScript IV does not display the G → T mutation as its major variation type, the dNTP/dATP ratio change did not show any improvements on its corresponding variation signature at this AP site generated from 18S rRNA m^7^G1639 (Figure S1G).

### m^7^G-quant-seq Detects Human tRNA m^7^G46 in High Variation Ratios

However, we found that the elevation of the dATP/dNTP ratio in the RT buffer led to dramatic RT stops at internal m^7^G sites when using any of these 5 RTs, although the adjustment in the dATP/dNTP ratio could induce a higher variation ratio for internal m^7^G detection. The RT stop caused by the higher dATP amount could impact the read-through of RNA fragments generated from tRNA. We previously found that the wild-type HIV RT with 1 mM dNTP could work very well on read-through of human tRNAs and reveal multiple tRNA methylations (such as m^1^A, m^3^C, m^1^G, m^2^_2_G) as misincorporation signatures in one pot in DAMM-seq.^27^ We decided to focus on the “1 mM dNTP” condition in m^7^G-quant-seq.

**Figure 2:**
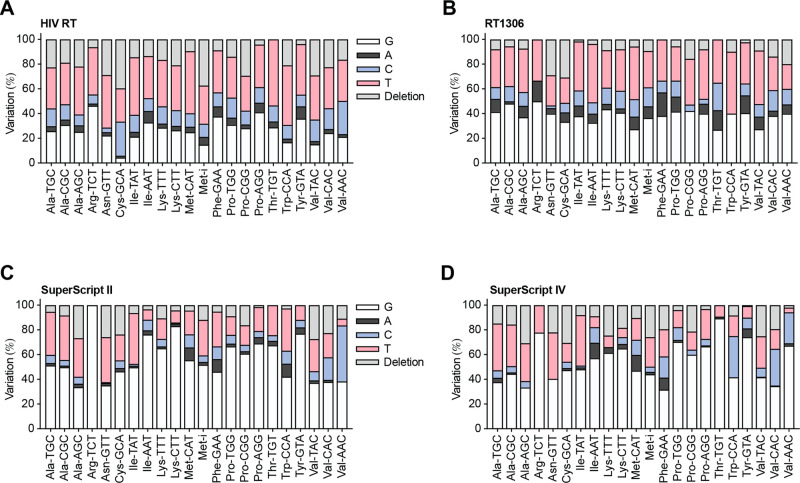
**m**^**7**^**G-quant-seq detects 22 tRNA m**^**7**^**G sites in human cytoplasmic tRNAs, as a mixed pattern of mutations and deletion.** (A) The variation signatures at the AP site generated from HeLa tRNA m^7^G46 under m^7^G-quant-seq treatment with wild-type HIV RT and 1 mM dNTP. (B) The variation signatures at the AP site generated from HeLa tRNA m^7^G46 under m^7^G-quant-seq treatment with engineered RT1306 and 1 mM dNTP. (C) The variation signatures at the AP site generated from HeLa tRNA m^7^G46 under m^7^G-quant-seq treatment with SuperScript II RT and 1 mM dNTP. (D) The variation signatures at the AP site generated from HeLa tRNA m^7^G46 under m^7^G-quant-seq treatment with SuperScript IV RT and 1 mM dNTP.

We performed the standard m^7^G-quant-seq protocol (Figure 1B) with four different RTs (under 1 mM dNTP) and sequenced each tRNA library at ∼10-20 M read depth. We indeed successfully detected strong signals for internal m^7^G sites in 22 human tRNAs, at a range of 54–96% total variation rates when using the wild-type HIV RT (Figure 2A), in which all tRNA m^7^G sites displayed a mixed variation pattern of mutations and deletions. These results are much better than those in m^7^G-seq, in the absence of biotin pulldown enrichment.^20^ We also screened the engineered RT1306^25^ under 1 mM dNTP and observed 50–73% total variation rates (Figure 2B), which are notably lower than the ratios in wild-type HIV RT. Although SuperScript II RT and SuperScript IV RT gave high variation ratios at 18S rRNA m^7^G site (Figure 1C), these two RTs displayed poor variation rates at all tRNA m^7^G sites (Figure 2C,D). Based on these data from tRNA m^7^G methylomes, we conclude that the wild-type HIV RT is most suitable for the tRNA m^7^G methylation measurement.

We noticed that, when using wild-type HIV RT, the pattern of misincorporation and deletion at the m^7^G46 site are different in 22 tRNAs from HeLa and HEK293T cells (Figure 2A), which can be attributed to sequence context difference around the m^7^G46 site. tRNA m^7^G46 is deposited in several different 5-mer motif contexts, such as AG(m^7^G)CC, AG(m^7^G)CU, AG(m^7^G)GU, AG(m^7^G)UA, AG(m^7^G)UC, AG(m^7^G)UU, and GG(m^7^G)UC; sequence differences contribute to different misincorporation and deletion ratios. While there is no notable difference in other RNA modifications around the m^7^G46 site (Figure S2), the sequence context beyond the 5-mer motif may also contribute to misincorporation and deletion changes during RT using HIV RT enzyme.

### m^7^G-quant-seq Calibration Curves to Determine m^7^G Stoichiometry

The internal m^7^G at position 1639 of 18S rRNA and position 46 of cytoplasmic tRNAs possess eight motif contexts, including AG(m^7^G)GA, AG(m^7^G)CC, AG(m^7^G)CU, AG(m^7^G)GU, AG(m^7^G)UA, AG(m^7^G)UC, AG(m^7^G)UU, and GG(m^7^G)GC. The synthetic challenges make it difficult to build RNA oligo probes that carry internal m^7^G within all these motifs. Instead, we synthesized RNA oligos containing an AP site at the expected m^7^G position because we are measuring the AP sites generated from m^7^G.

**Figure 3:**
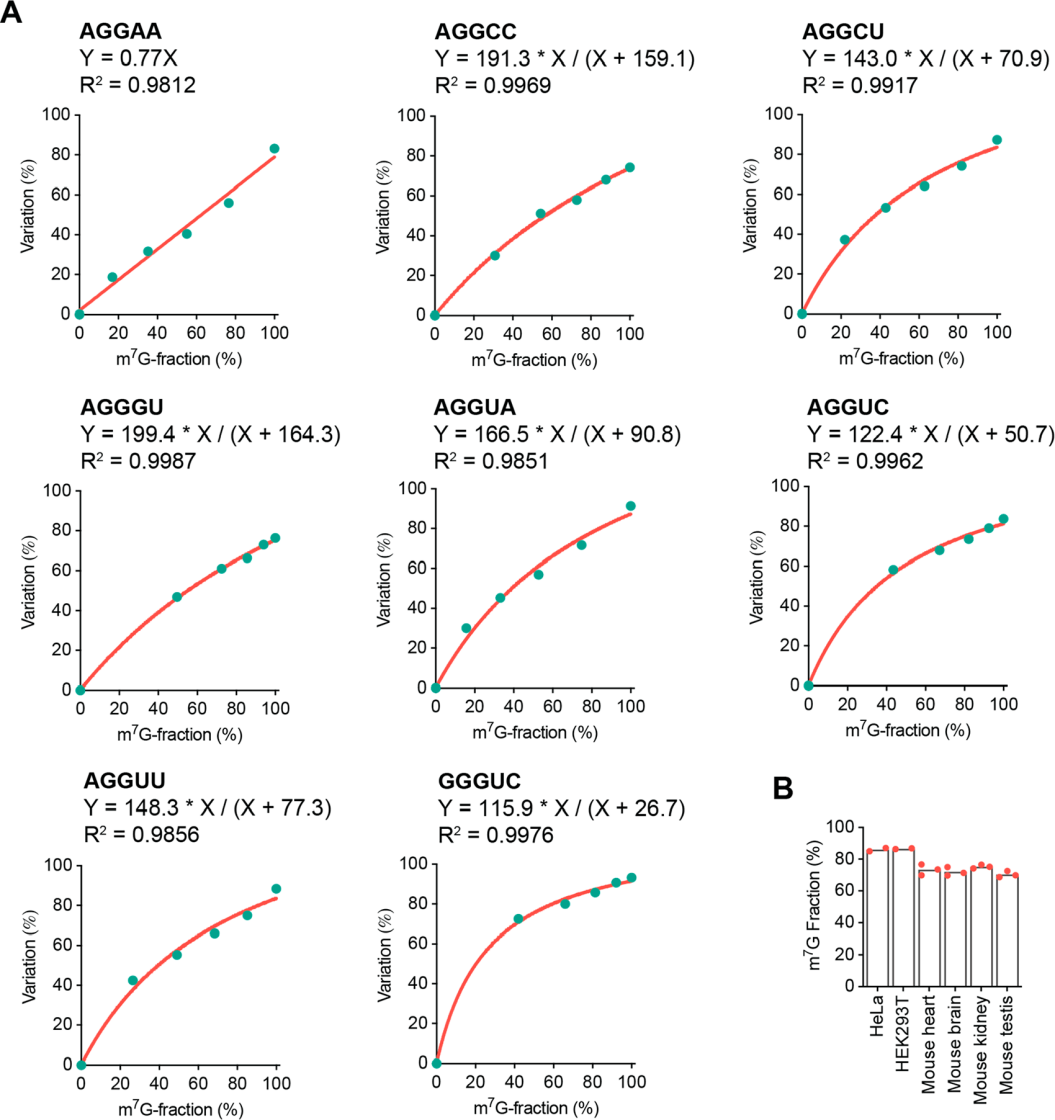
**m**^**7**^**G-quant-seq calibration curves for motif contexts around internal m**^**7**^**G sites in human tRNAs and 18S rRNA.** (A) Representative sequence-context-dependent calibration curves (total variation rate vs methylation fraction) for m^7^G fraction quantification, including eight major motif contexts around internal m^7^G sites in human rRNA and tRNA, under m^7^G-quant-seq treatment with HIV RT (1 mM dNTP). (B) The m^7^G methylation fractions of 18S rRNA m^7^G1639 in HeLa cells, HEK293T cells, mouse heart, mouse brain, and mouse kidney, respectively.

We mixed oligo probes containing NN(AP-site)NN and NNGNN (as controls) to plot calibration curves for all sequence contexts. We obtained either linear curves or hyperbola curves for the eight motif contexts around internal m^7^G sites in human rRNA and tRNA (Figure 3A). For instance, based on the calibration curve of AG(m^7^G)AA, the fraction of the m^7^G site in 18S rRNA was calculated to be at least 85% in HeLa and HEK293T cells (Figure 3B), generally consistent with those measured by mass spectrometry.^30^ The methylation fraction of m^7^G in 18S rRNA seems to be slightly lower in mouse tissues, compared with cultured cells (Figure 3B). Notably, m^7^G-quant-seq only detected one m^7^G candidate site at position 1639 among hundreds of guanosine sites in HeLa 18S rRNA (Figure S3A), when applying several cutoffs for internal m^7^G detection, including: (1) variation (misincorporation and deletion) ratio above 5% in m^7^G-quant-seq libraries; (2) variation ratio below 5% in “Input” libraries; (3) total reads coverage depth above 20 in both m^7^G-quant-seq and “Input” libraries; (4) variation ratio in m^7^G-quant-seq libraries greater than fivefold over that in “input” libraries; (5) variation ratio in m^7^G-quant-seq libraries greater than fivefold over the background in any given sequence motif (defined as the variation rates detected from RNA probes containing unmodified NNGNN after m^7^G-quant-seq treatment). Additionally, all misincorporation and deletion signatures must occur at the internal positions of the reads, instead of reads end.

### m^7^G-quant-seq Estimates the Stoichiometry of Internal m^7^G Sites in 22 Cytoplasmic tRNAs

**Figure 4:**
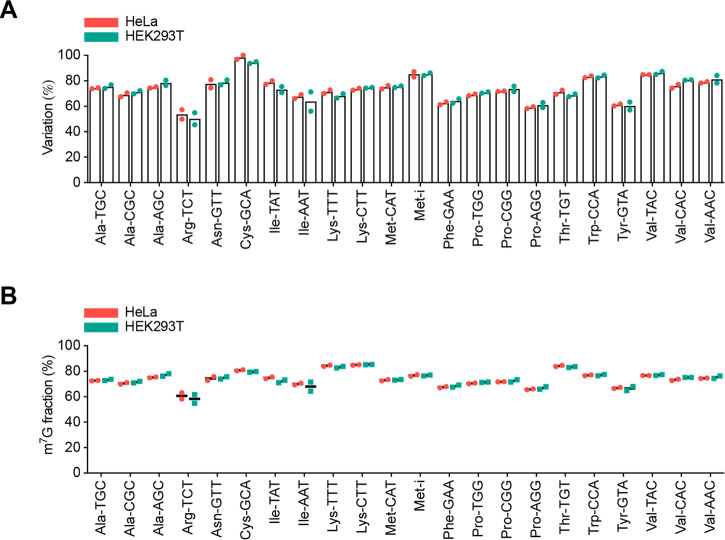
**m**^**7**^**G-quant-seq measures the stoichiometry of internal m**^**7**^**G sites in 22 human tRNAs.** (A) The total variation rate at the AP site generated from m^7^G46 of 22 cytoplasmic tRNAs in HeLa and HEK293T cells, under m^7^G-quant-seq treatment with HIV RT. Data dots from two biological replicates are shown. (B) The m^7^G methylation fraction at m^7^G46 of 22 cytoplasmic tRNAs in HeLa and HEK293T cells, respectively. Data dots from two biological replicates are shown.

With the calibration curves in hand, we were able to measure the methylation fractions at internal m^7^G sites on human cytoplasmic tRNAs. We applied m^7^G-quant-seq to cellular small RNAs (<200 nt) from HeLa and HEK293T cells. In both cell lines, we observed strong variation signatures at internal m^7^G sites in 22 cytoplasmic tRNAs, at a range of 50–97% total variation rates (Figure 4A). Internal m^7^G methylomes were not detected in human mitochondrial tRNAs, which is consistent with the findings in m^7^G-seq.^20^

Based on the calibration curves of the seven major m^7^G motifs in human tRNA, we calculated the modification fractions of these m^7^G sites according to the corresponding variation ratios. We found that the m^7^G stoichiometry in human tRNAs ranges around 60–85% at least, and most tRNA m^7^G sites display at least 70% methylation fraction in these two human cell lines (Figure 4B). Highly consistent results indicate a superb performance of the current protocol. This method should allow accurate determination of m^7^G stoichiometry changes under different stress or different cellular contexts as well as in other RNA species.

Although TRAC-seq is based on RT truncation signatures and cannot provide stoichiometry information on m^7^G methylation, it detected 25, 22, 17, 21, and 19 tRNA m^7^G sites in LNZ308, HuCCT1, MHCC97H, NBL, and ESCC cells, respectively (Figure S3B).^14−17,31^ In m^7^G-quant-seq and TRAC-seq, m^7^G sites on 11 tRNAs are consistent among all aforementioned human cell lines, including Ala-TGC, Arg-TCT, Cys-GCA, Lys-TTT, Lys-CTT, Met-CAT, Phe-GAA, Thr-TGT, Trp-CCA, Val-CAC, and Val-AAC. However, m^7^G sites on other tRNAs showed more dynamic changes in different cell lines, indicating differential methylation fractions at these m^7^G tRNA sites in different cancer cells (Figure S3B).

## Discussion

We report here m^7^G-quant-seq based on the chemistry principle of RT-misincorporation-based m^7^G-seq ^20^. m^7^G-quant-seq employs new conditions to achieve highly efficient reduction and depurination at the internal m^7^G site, leading to the almost complete conversion of internal m^7^G sites into RNA abasic sites. We screened RT enzymes and RT conditions to achieve maximum mutation and deletion changes from the RNA abasic sites. The variation signals in the new procedure increased by at least twofold compared to the previous procedure. We also include a brief and fast protocol for library construction, which starts with ∼200 ng of RNA (Supporting Information). The 4 h KBH_4_ reduction step (at room temperature) and the 4 h acidic depurination step (at 45 °C) are easy to handle and can be robustly repeated.

We observed that, under multiple different RTs, an RNA abasic site induces a mixed variation pattern during reverse transcription, including G → A or C or T mutations and meanwhile deletions (Figure 1C). In this study, we conducted a comprehensive study on variation patterns at RNA abasic sites generated from internal m^7^G sites. In addition to m^7^G mapping, this method could potentially benefit future research on RNA abasic sites and abasic sites generated from other RNA modifications as well. In HeLa and HEK293T cells, m^7^G-quant-seq detected internal m^7^G sites in 22 human cytoplasmic tRNAs with high variation ratios (Figures 2A and 4A). Using proper calibration curves these high variation rates enabled us to successfully estimate the corresponding m^7^G methylation stoichiometry (Figure 4B), without any pulldown enrichment of these variation signatures.

In summary, m^7^G-quant-seq could be broadly applied to quantitatively monitor tRNA m^7^G methylation level change in diverse biological processes, such as gene knockdown, cellular stress, heat shock, cancer progression, etc. Considering the METTL1-mediated tRNA m^7^G regulates tumorigenesis and cancer progression in many cancer types, m^7^G-quant-seq could potentially facilitate a series of future investigations on METTL1 functions in cancer biology and cancer therapy.

## Supporting Information Available

A complete description of Methods for cell culture, RNA isolation, calibration curves, m^7^G-quant-seq library construction, and sequencing data analysis (PDF)
