# Supplementary material for: m7G-quant-seq: Quantitative Detection of RNA Internal N7-Methylguanosine
Source: ACS Chem Biol. 2022 Nov 18;17(12):3306–12. doi: 10.1021/acschembio.2c00792 (PMC9764283; doi:10.1021/acschembio.2c00792)
Supplement: Supplementary file 1 — cb2c00792_si_001.pdf [file cb2c00792_si_001.pdf]

Supporting Information

m⁷G-quant-seq: Quantitative Detection of RNA Internal N^7-Methylguanosine.

Li-Sheng Zhang [†, ‡,#,*], Cheng-Wei Ju [₴,#], Chang Liu [†,#], Jiangbo Wei [†], Qing Dai [†], Li Chen [†], Chang Ye [†], Chuan He [†,*]

[†] Department of Chemistry, The University of Chicago; Howard Hughes Medical Institute, The University of Chicago; Chicago, Illinois 60637, United States.

[‡] Division of Life Science, Department of Chemistry, The Hong Kong University of Science and Technology, Clear Water Bay, 999077, Kowloon, Hong Kong SAR, China.

[₴] Pritzker School of Molecular Engineering, The University of Chicago; Chicago, Illinois 60637, United States.

* Corresponding author: chuanhe@uchicago.edu, lszhang@uchicago.edu.

[#] These authors contributed equally.

Table of Content

Title page 1
Table of content 2
Supporting Figure S1 3
Supporting Figure S2 4
Supporting Figure S3 5
Methods...6
Cell culture...6
RNA isolation..6
Library construction protocol...6
Data analysis..9
Calibration curves...10
Data availability...11
Supporting Table S1 12
Supporting Table S2 13

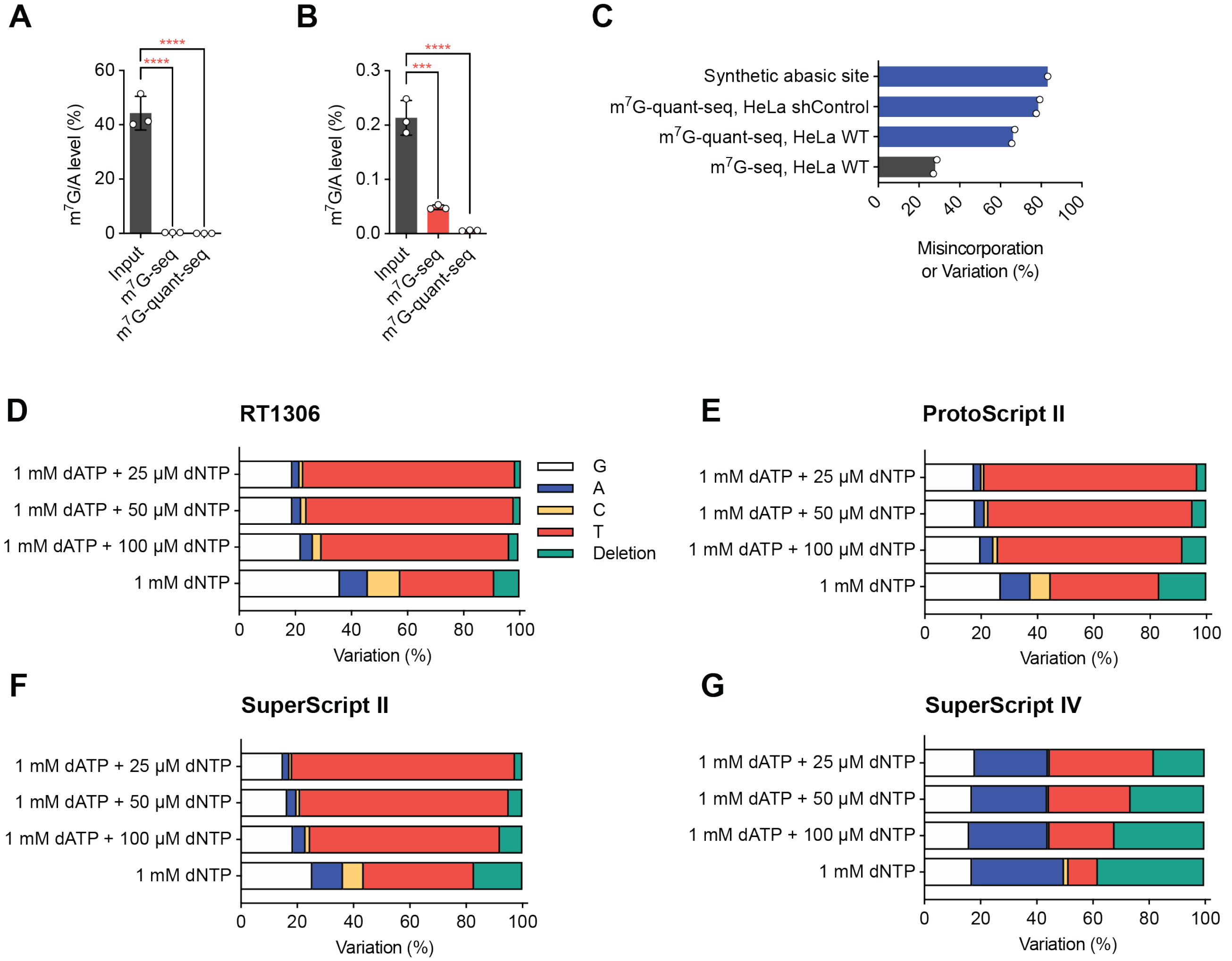

Figure S1. (A) m^{7}G/A levels in synthetic RNA oligo after m^{7}G-seq $NaBH_4$ treatment and m^{7}G-quant-seq KBH_4 treatment, versus the untreated input, revealed by LC-MS/MS. n = 3, technically independent replicates. (B) m^{7}G/A levels in fragmented HeLa total RNA after m^{7}G-seq $NaBH_4$ treatment and m^{7}G-quant-seq KBH_4 treatment, versus the untreated input, revealed by LC-MS/MS. n = 3, biologically independent replicates. (C) The misincorporation or variation ratios at 18S rRNA m^{7}G1639, which were uncovered by m^{7}G-seq and m^{7}G-quant-seq with total RNA isolated from wild-type or shControl HeLa cells. (D) The variation signatures at the AP site generated from HeLa 18S rRNA m^{7}G1639 under m^{7}G-quant-seq treatment with engineered RT1306 and adjusted dNTP/dATP ratios. (E) The variation signatures at the AP site generated from HeLa 18S rRNA m^{7}G1639 under m^{7}G-quant-seq treatment with ProtoScript II RT and adjusted dNTP/dATP ratios. (F) The variation signatures at the AP site generated from HeLa 18S rRNA m^{7}G1639 under m^{7}G-quant-seq treatment with SuperScript II RT and adjusted dNTP/dATP ratios. (G) The variation signatures at the AP site generated from HeLa 18S rRNA m^{7}G1639 under m^{7}G-quant-seq treatment with SuperScript IV RT and adjusted dNTP/dATP ratios.

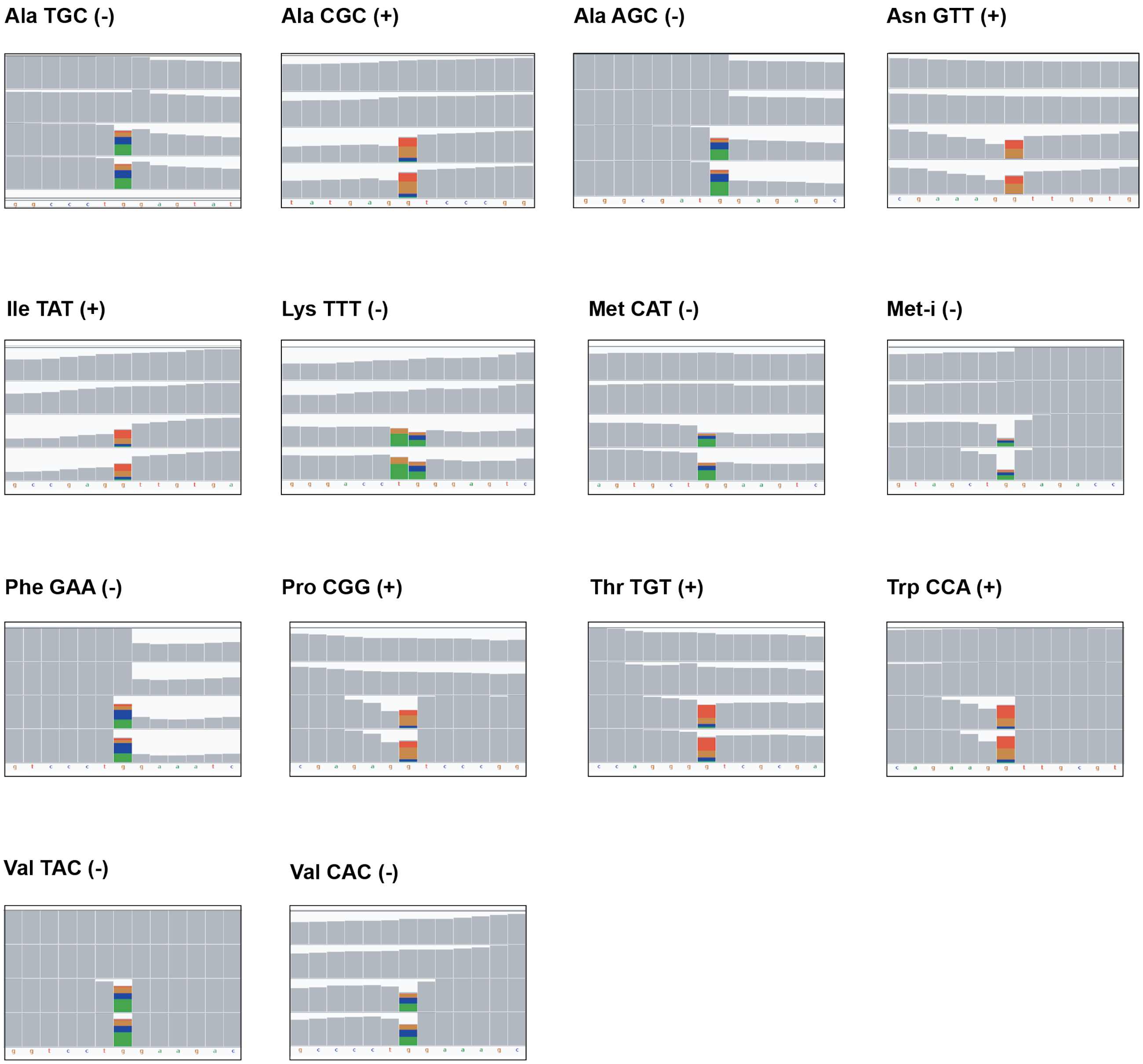

Figure S2. IGV plot of internal m^7G_{46} site in representative tRNAs from HeLa cells. The upper two rows in each panel are from 'input' samples, n = 2, biologically independent replicates; the lower two rows in each panel are from 'm^7G-quant-seq' samples, n = 2, biologically independent replicates. For tRNA labeled with (+), these tRNAs locate on sense strand in human genome, in which A, C, G, and U are marked by green, blue, brown, and red colors, respectively. For tRNA labeled with (-), these tRNAs locate on antisense strand in human genome, in which A, C, G, and U are marked by red, brown, blue, and green colors, respectively.

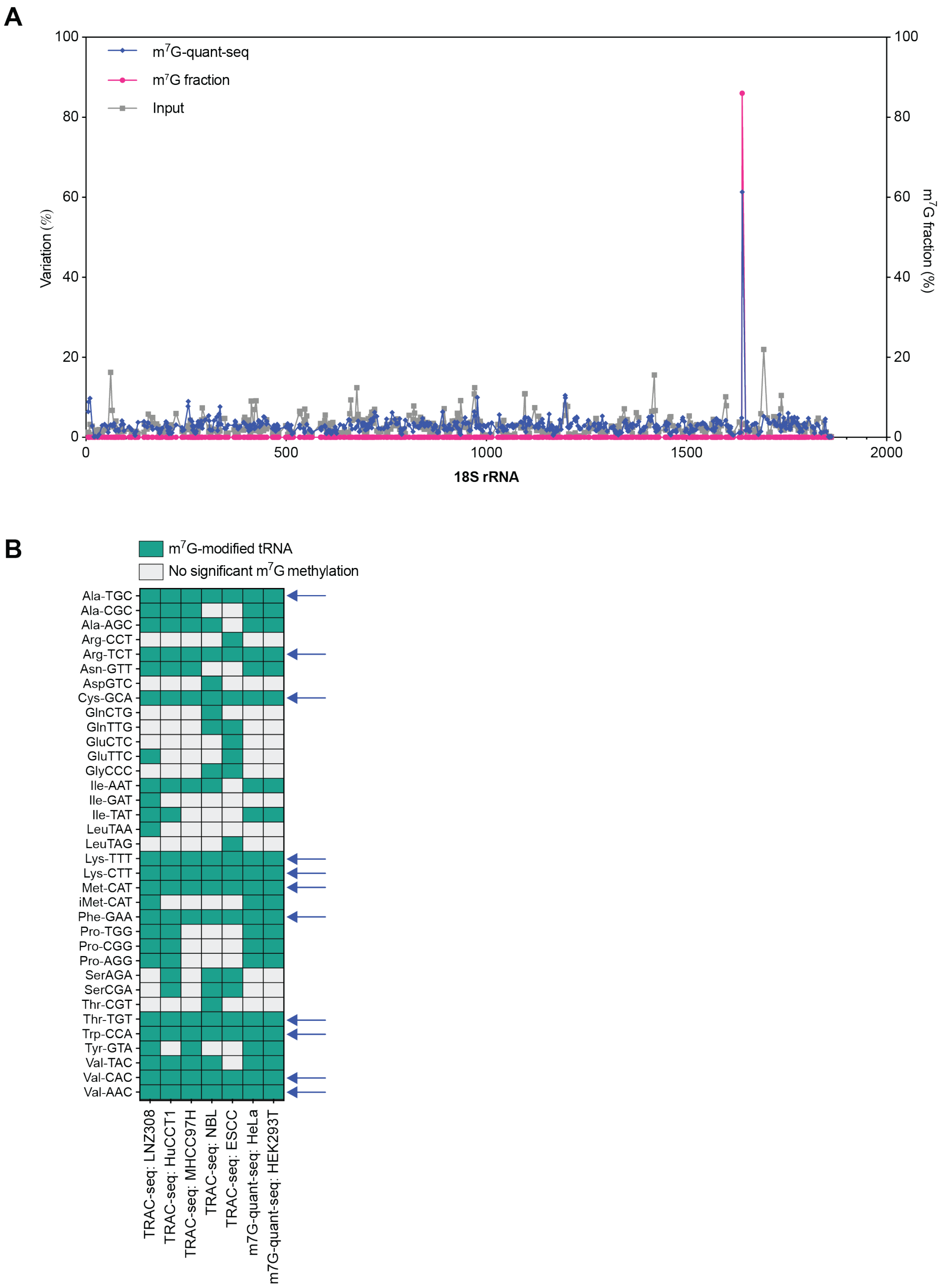

Figure S3. (A) The variation ratios in m^{7}G-quant-seq, variation ratios in 'Input' (with any chemical treatment), and estimated m^{7}G methylation fraction at all guanosine sites in HeLa 18S rRNA. (B) The identified m^{7}G sites in tRNA from different human cell lines, for a comparison of m^{7}G detection in m^{7}G-quant-seq and TRAC-seq.

Methods

Cell culture

HeLa and HEK 293T cell lines were purchased from the American Type Culture Collection (ATCC). The HeLa cell line was grown in DMEM medium (Gibco, 11965) supplemented with 10% v/v FBS and 1% penicillin/streptomycin (Gibco). The HEK 293T cell line was maintained in DMEM (Gibco, 11995) with 10% FBS and 1% penicillin/streptomycin. Cells were cultured at 37 °C with 5.0% CO2 in a Heracell VIOS 160i incubator (Thermo Scientific).

Small RNA isolation

Cellular total RNA was isolated with TRIzol reagent (Invitrogen) following the manufacturer's protocol by isopropanol precipitation. The small RNA fraction (size < 200 nt) was further extracted from the purified total RNA using the mirVana miRNA Isolation Kit (AM1560, Invitrogen).

m^{7}G-quant-seq

RNA Fragmentation:

The starting amount of RNA could be 200 ng before fragmentation. Then RNA Fragmentation Reagent (Invitrogen, AM8740) was used as 15X (add 1.0 µL buffer into 14 µL of RNA), which is originally 10X. The heating condition is 70 °C for 14 min, followed by Oligo Clean and Concentrator kit (OCC). Elute RNA to 22 µL (twice, 11 µL each time). Because we expect to capture the real methylation fraction of the internal m^{7}G site in tRNA, we did not perform any AlkB demethylation treatment to erase m^{1}A, m^{3}C, or m^{1}G methylations on tRNA; in this way, it is hard for HIV RT to read through the full-size tRNA. Fragmenting tRNAs into 30-50 nt will facilitate HIV RT to read through the shorter fragments generated from tRNAs, reflecting the actual m^{7}G methylation fraction *via* variation signatures.

End repair:

Prepare the end repair reaction as follow:

Reagents	Volume (µL)
RNA	~200 ng
10X PNK reaction buffer	3
T4 PNK	3
SUPERase•In™	1.5

Prepare a stock containing RNA + 10X PNK buffer + T4 PNK + SUPERase•In™ with a final volume of 30 µL. Mix the stock well and incubate at 37 °C for 45 min. Recover RNA with Oligo Clean and Concentrator kit (OCC) and elute to 10 µL with RNase-free water.

Adaptor ligation:

Mix 1.0 µL 20 µM 3' linker (5′rApp-NNNNNAGATCGGAAGAGCGTCGTG-3SpC3) with repaired 10 µL RNA fragments and incubate the mixture at 70 °C for 2 min for denaturation. Immediately move onto ice. Please pay attention to the actual adaptor sequences in Table S1. Prepare the ligation reaction as follow:

Reagents	Volume (µL)
RNA and linker mixture	11
10X T4 RNA Ligase Reaction Buffer	2.5
50% PEG8000	7.5
SUPERase•In™	1
T4 RNA ligase 2 truncated KQ (NEB)	2
H_2O	1

Mix everything well before adding T4 RNA ligase 2 truncated KQ. Then add 2.0 µL T4 RNA ligase 2 truncated KQ and mix well again. Incubate the reaction at 25 °C for 2 hours, followed by 16 °C for 10 hours.

Then add 23 µL RNase-free water to dilute the reaction mixture. Add 2.0 µL 5-deadenylase, mix well/incubate at 30 °C for 30 min; then add 1.0 µL RecJf, mix well/incubate at 37 °C for another 30 min. Recover RNA with RNA Clean and Concentrator kit (RCC) and elute to 12 µL with RNase-free water.

Save 2 µL as 'input'; use the rest for the following steps. Safe stop point: -80C for 1 week.

Reduction:

Put the eluted RNA in 10 µL RNase-free water. Prepare a fresh 1.0M KBH_4 buffer in RNase-free water. Add 40 µL KBH_4 buffer into RNA and mix well. Incubate at room temperature for 4 hours (avoid the light). Recover RNA with RNA Clean and Concentrator kit (RCC) and elute to 45 µL with RNase-free water.

Generation of RNA abasic sites:

Add 5.0 µL 1.0M NaOAc/AcOH buffer (pH 2.9) into the purified RNA and mix well. Incubate with heating for 4 hours (avoid the light). Recover RNA with Oligo Clean and Concentrator kit (OCC) and elute

to 10 µL with RNase-free water. 1.0M NaOAc/AcOH buffer was prepared with 3.3 mL 3M NaOAc (pH 5.5, Invitrogen™, AM9740) and 6.7 mL acetic acid (Fisher BioReagents, BP2401-500).

Reverse transcription:

Mix 1.0 µL 2 µM RT-primer (5′-ACACGACGCTCTTCCGATCT-3′) with 10 µL depurinated RNA fragments and incubate the mixture at 65 °C for 2 min for denaturation. Immediately move onto ice. Prepare the ligation reaction as follow:

Reagents	Volume (µL)
RNA/primer mixture	11
10X AMV RT Reaction Buffer	2
dNTP (10 mM)	2
RNase Out	0.5
H_2O	2.5
HIV RT	2

Mix everything well before adding HIV RT. Then add 2.0 µL HIV RT and mix well again. Incubate the reaction at 37 °C for 1.5 hours.

Then add 1.0 µL RNase H (NEB) into the reaction mixture. Mix well/incubate at 37 °C for 20 min. Then 70 °C for 5 min to denature.

Recover RNA with Oligo Clean and Concentrator kit (OCC) and elute to 10 µL with RNase-free water. Safe stop point: -80C for 1 week.

cDNA 3′-ligation:

Mix 1.0 µL 50 µM cDNA 3'-adapter (5′Phos-NNNNNAGATCGGAAGAGCACACGTCTG-3SpC3) with 10 µL cDNA and incubate the mixture at 75 °C for 2 min for denaturation. Immediately move onto ice. Please pay attention to the actual adaptor sequences in Table S1. Prepare the ligation reaction as follow:

Reagents	Volume (µL)
cDNA/adapter mixture	11
10X T4 RNA Ligase Reaction Buffer	3
50% PEG8000	15

10mM ATP	3
T4 RNA Ligase 1	1

Mix everything well before adding T4 RNA Ligase 1. Then add 1.0 μL T4 RNA Ligase 1 and mix well again. Add ligase enzyme to one sample followed by mixing this sample immediately. Mix each sample one by one. Do not add ligase enzyme to all samples and then start mixing everything.

Incubate the reaction at 25 °C for 12 hours.

PCR Amplification:

After the 12-hour cDNA ligation, heat at 65 °C for 5 min to denature. Purify the cDNA by DNA Clean & Concentrator-5. Elute cDNA with 20 μL DNase-free water. Use 4 μL per sample for each PCR amplification.

Identification of variation signatures in m^7G-quant-seq

The sequencing data were all trimmed with the cutadapt tool to remove adapters and low-quality reads (lengths shorter than 20 bp). PCR duplicates were removed with the BBMap tool (https://sourceforge.net/projects/bbmap/), random barcodes at reads end were trimmed, and low-quality reads were removed using the cutadapt tool. The remaining reads were aligned to the human genome (hg38) using Tophat2 (version 2.1.1) and bowtie2 (version 2.3.5.1) allowing a maximum of three mismatches. The generated .bam files were split into positive and negative strands and sorted using Samtools. Sequence variants were identified by measuring the base composition at each position using fine-tuned bam-readcount (https://github.com/genome/bam-readcount). The generated bam-readcount output results were parsed and analyzed to calculate the misincorporation/deletion ratio at each abasic site generated from internal m^7G site, followed by confirmation using direct visualization through IGV software (https://software.broadinstitute.org/software/igv/).

The m^7G candidate sites must satisfy the criteria shown below:

(1) variation (misincorporation and deletion) ratio above 5% in m^7G-quant-seq libraries; (2) variation ratio below 5% in 'Input' libraries; (3) total reads coverage depth above 20 in both m^7G-quant-seq and 'Input' libraries; (4) variation ratio in m^7G-quant-seq libraries is > 5-fold over that in 'input' libraries; (5) variation ratio in m^7G-quant-seq libraries is > 5-fold over the background in any given sequence motif (defined as the variation rates detected from RNA probes containing unmodified NNGNN after m^7G-quant-seq treatment). Additionally, all misincorporation and deletion signatures must occur at the internal positions of the reads, instead of reads end.

Note that the estimated m^7G methylation fraction (in m^7G -quant-seq) serves as the minimum value of methylation stoichiometry at the methylated site, because the calibration curves were built with NN(AP-site)NN oligos instead of NN(m^7G)NN oligos. The actual m^7G methylation fraction could be a little higher than the estimated methylation stoichiometry in m^7G -quant-seq, due to the fact that it is hard to achieve 100% chemical conversion at m^7G site in all motif contexts.

Calibration curves for m^7G-quant-seq

The 31-mer RNA probe GAACGNN/irSp/NNUUCCCAGUACGUGAUGCCAAU (from IDT) was used as "100% AP-site" standard. The RNA oligo GAACGNNGNNUUCCCAGUACGUGAUGCCAAU (from IDT) was used as "0% AP-site". Then the "100% AP-site" and "0% AP-site" standard were mixed and generated 6 oligo mixtures with different modification fractions, as 100%, 80%, 60%, 40%, 20% and 0% AP-site.

For 200 ng of each set of mixed RNA oligos (as 100%, 80%, 60%, 40% ,20% and 0% AP-site), proceed with RNA 3'-ligation directly. Mix 1.0 µL 20 µM 3' linker (5′rApp-NNNNNAGATCGGAAGAGCGTCGTG-3SpC3) with 10 µL RNA oligos and incubate the mixture at 70 °C for 2 min for denaturation. Immediately move onto the ice. Then add 2.5 µL 10X T4 RNA Ligase Reaction Buffer, 7.5 µL 50% PEG8000, 1 µL SUPERNase-In, and 1 µL H_2O. Mix everything well before adding T4 RNA ligase 2 truncated KQ. Then add 2.0 µL T4 RNA ligase 2 truncated KQ and mix well again. Incubate the reaction at 25 °C for 2 hours, followed by 16 °C for 10 hours. Then add 23 µL RNase-free water to dilute the reaction mixture. Add 2.0 µL 5-deadenylase, mix well/incubate at 30 °C for 30 min; then add 1.0 µL RecJf, mix well/incubate at 37 °C for another 30 min. Recover RNA with RNA Clean and Concentrator kit (RCC) and elute to 10 µL with RNase-free water.

Mix 1.0 µL 2 µM RT-primer (5′-ACACGACGCTCTTCCGATCT-3′) with 2 µL ligated RNA oligos and incubate the mixture at 65 °C for 2 min for denaturation. Immediately move onto the ice. Then add 2 µL 10X AMV RT Reaction Buffer, 2 µL 10mM dNTP, 0.5 µL RNaseOut, and 10.5 µL H_2O. Mix everything well before adding HIV RT. Then add 2.0 µL HIV RT and mix well again. Incubate the reaction at 37 °C for 1.5 hours. After RT, add 1.0 µL RNase H (NEB) into the reaction mixture. Mix well/incubate at 37 °C for 20 min. Then 70 °C for 5 min to denature. Recover RNA with Oligo Clean and Concentrator kit (OCC) and elute to 10 µL with RNase-free water.

Mix 1.0 µL 50 µM cDNA 3'-adapter (5′Phos-NNNNNAGATCGGAAGAGCACACGTCTG-3SpC3) with 10 µL cRNA and incubate the mixture at 75 °C for 2 min for denaturation. Immediately move onto the ice. Then add 3 µL 10X T4 RNA Ligase Reaction Buffer, 15 µL 50% PEG8000, and 3 µL 10mM ATP. Mix everything well before adding T4 RNA Ligase 1. Then add 1.0 µL T4 RNA Ligase 1 and mix well again. Add ligase enzyme to one sample followed by mixing this sample immediately. Mix each sample one by one.

Incubate the reaction at 25 °C for 12 hours. After the 12-hour cDNA ligation, heat at 65 °C for 5 min to denature. Purify the cDNA by DNA Clean & Concentrator-5. Elute cDNA with 20 μL DNase-free water. Use 4 μL per sample for each PCR amplification.

Data availability

The sequencing data listed in Table S1 are available in the Gene Expression Omnibus database under the accession number GSE209646. All other data supporting the findings of this study are available from the corresponding author upon reasonable request.

Table S1 Sample information for high-through sequencing in this study.

Experiment	Samples/replicates	Reads#
"Treated" samples for m^7G-quant-seq to study the variation patterns at HeLa 18S rRNA m^7G1639, under five RTs and different dNTP/dATP ratios.<br><br>RNA 3'-adaptor:<br>5'rApp-NNNNN AGATCGGAAGAGCGTCGTG-3SpC3<br><br>cDNA 3'-adaptor:<br>5'Phos-**NNNNNN** AGATCGGAAGAGCA CACGTCTG-3SpC3	HeLa_Total-RNA_HIV-RT_1mM-dNTP	16141020
	HeLa_Total-RNA_HIV-RT_100uM-dNTP_1mM-dATP	20636416
	HeLa_Total-RNA_HIV-RT_50uM-dNTP_1mM-dATP	14878546
	HeLa_Total-RNA_HIV-RT_25uM-dNTP_1mM-dATP	16306827
	HeLa_Total-RNA_RT1306_1mM-dNTP	16842442
	HeLa_Total-RNA_RT1306_100uM-dNTP_1mM-dATP	21461811
	HeLa_Total-RNA_RT1306_50uM-dNTP_1mM-dATP	17791752
	HeLa_Total-RNA_RT1306_25uM-dNTP_1mM-dATP	13265386
	HeLa_Total-RNA_ProtoScript-II_1mM-dNTP	18836930
	HeLa_Total-RNA_ProtoScript-II_100uM-dNTP_1mM-dATP	20122916
	HeLa_Total-RNA_ProtoScript-II_50uM-dNTP_1mM-dATP	18798320
	HeLa_Total-RNA_ProtoScript-II_25uM-dNTP_1mM-dATP	18842670
	HeLa_Total-RNA_SuperScript-II_1mM-dNTP	19325522
	HeLa_Total-RNA_SuperScript-II_100uM-dNTP_1mM-dATP	15369495
	HeLa_Total-RNA_SuperScript-II_50uM-dNTP_1mM-dATP	21160520
	HeLa_Total-RNA_SuperScript-II_25uM-dNTP_1mM-dATP	16375172
	HeLa_Total-RNA_SuperScript-IV_1mM-dNTP	22002048
	HeLa_Total-RNA_SuperScript-IV_100uM-dNTP_1mM-dATP	27735869
	HeLa_Total-RNA_SuperScript-IV_50uM-dNTP_1mM-dATP	22923868
	HeLa_Total-RNA_SuperScript-IV_25uM-dNTP_1mM-dATP	24853810
Calibration curves for estimating internal m^7G methylation fractions, under HIV RT and 1mM dNTP.<br>RNA 3'-adaptor:<br>5'rApp-NNNNN AGATCGGAAGAGCGTCGTG-3SpC3<br>cDNA 3'-adaptor:<br>5'Phos-NNNNN AGATCGGAAGAGCA CACGTCTG-3SpC3	CalibrationCurves_HIV-RT_0%_AP-site	15560359
	CalibrationCurves_HIV-RT_20%_AP-site	16465341
	CalibrationCurves_HIV-RT_40%_AP-site	15798437
	CalibrationCurves_HIV-RT_60%_AP-site	15521390
	CalibrationCurves_HIV-RT_80%_AP-site	10204722
	CalibrationCurves_HIV-RT_100%_AP-site	12452862
Duplicates for m^7G-quant-seq "Treated" and "Input" samples to reveal tRNA m^7G46 methylation fractions in HeLa and HEK293T cells, under HIV RT and 1mM dNTP.<br><br>RNA 3'-adaptor:<br>5'rApp-NNNNN AGATCGGAAGAGCGTCGTG-3SpC3<br>cDNA 3'-adaptor:<br>5'Phos-NNNNN AGATCGGAAGAGCA CACGTCTG-3SpC3	HeLa_SmallRNA_HIV-RT_Treated_rep1	21275083
	HeLa_SmallRNA_HIV-RT_Treated_rep2	24798655
	HEK293T_SmallRNA_HIV-RT_Treated_rep1	21344929
	HEK293T_SmallRNA_HIV-RT_Treated_rep2	20370688
	HeLa_SmallRNA_HIV-RT_Input_rep1	18618694
	HeLa_SmallRNA_HIV-RT_Input_rep2	21454705
	HEK293T_SmallRNA_HIV-RT_Input_rep1	21536302
	HEK293T_SmallRNA_HIV-RT_Input_rep2	18117600

Table S2 Variation signatures at all guanosine sites on human 18S rRNA.

rRNA	*Position*	*Motif*	*Variation rate in m^7G-quant-seq (%)*	*Variation rate in 'Input' (%)*	*Background variation rate (%) in a specific motif from NNGNN, after m^7G-quant-seq treatment*
18S	6	CTGGT	6.402	1.307	0.338
18S	7	TGGTT	8.857	1.162	1.856
18S	10	TTGAT	9.775	3.233	2.577
18S	16	CTGCC	2.852	2.819	6.599
18S	20	CAGTA	0.113	0.767	4.176
18S	23	TAGCA	0.263	1.806	2.183
18S	29	ATGCT	0.125	1.587	4.854
18S	33	TTGTC	0.719	1.972	2.079
18S	41	AAGAT	3.003	1.024	3.643
18S	47	AAGCC	3.271	1.813	4.412
18S	52	ATGCA	2.759	2.295	3.876
18S	56	ATGTC	1.262	2.491	5.270
18S	62	GAGTA	3.167	16.276	2.726
18S	66	ACGCA	2.881	6.781	10.722
18S	70	ACGGC	3.471	2.704	2.500
18S	71	CGGCC	2.872	2.634	0.813
18S	74	CCGGT	1.632	2.249	5.096
18S	75	CGGTA	1.729	4.099	2.384
18S	80	CAGTG	1.970	0.823	1.099
18S	82	GTGAA	1.609	1.964	3.093
18S	88	CTGCG	3.154	1.471	0.524
18S	90	GCGAA	4.154	2.236	2.219
18S	94	ATGGC	3.279	1.514	2.869
18S	95	TGGCT	1.626	1.516	1.397
18S	108	CAGTT	2.650	2.016	1.926
18S	113	ATGGT	2.135	0.296	2.203
18S	114	TGGTT	1.843	0.774	1.856
18S	122	TTGGT	0.950	0.400	2.811
18S	123	TGGTC	0.466	0.872	3.716
18S	126	TCGCT	1.511	1.540	9.829
18S	130	TCGCT	3.750	1.039	9.829
18S	145	TTGGA	3.990	1.329	3.889
18S	146	TGGAT	2.962	3.333	1.790
18S	153	CTGTG	2.560	2.081	1.541
18S	155	GTGGT	3.677	2.703	1.974
18S	156	TGGTA	3.605	5.788	1.994
18S	165	TAGAG	2.663	3.451	0.606
18S	167	GAGCT	1.969	4.988	1.250
18S	177	ATGCC	2.246	3.389	8.696
18S	180	CCGAC	2.050	2.765	4.167
18S	183	ACGGG	1.950	0.969	2.791
18S	184	CGGGC	1.033	1.170	3.486
18S	185	GGGCG	1.318	1.244	2.020
18S	187	GCGCT	1.758	1.116	2.963
18S	190	CTGAC	2.310	1.225	2.013
18S	200	TCGCG	3.932	2.841	1.815
18S	202	GCGGG	3.603	1.443	5.556

18S	203	CGGGG	2.831	0.517	5.217
18S	204	GGGGG	2.446	0.516	6.000
18S	205	GGGGG	1.655	0.469	6.000
18S	206	GGGGG	1.552	0.374	6.000
18S	207	GGGGA	1.018	0.280	0.769
18S	208	GGGAT	0.938	0.837	1.140
18S	211	ATGCG	2.331	1.372	1.871
18S	213	GCGTG	0.965	3.118	2.277
18S	215	GTGCA	1.162	2.414	2.222
18S	225	CAGAT	3.152	5.958	2.605
18S	240	CCGGT	2.451	1.519	5.096
18S	241	CGGTC	1.651	1.861	5.469
18S	245	CAGCC	1.857	1.054	4.555
18S	255	CCGGC	7.745	1.918	2.588
18S	256	CGGCC	8.977	2.906	0.813
18S	261	CCGGC	4.477	4.283	2.588
18S	262	CGGCC	4.352	2.840	0.813
18S	265	CCGGG	3.976	1.328	5.769
18S	266	CGGGG	2.868	0.780	5.217
18S	267	GGGGG	1.807	1.039	6.000
18S	268	GGGGG	2.527	1.127	6.000
18S	269	GGGGC	2.857	1.569	2.646
18S	270	GGGCG	2.640	2.012	2.020
18S	272	GCGGG	1.159	1.142	5.556
18S	273	CGGGC	1.178	0.918	3.486
18S	274	GGGCG	1.040	1.797	2.020
18S	276	GCGCC	1.523	1.779	3.614
18S	279	CCGGC	3.169	1.116	2.588
18S	280	CGGCG	3.976	1.642	1.391
18S	282	GCGGC	2.780	3.086	1.227
18S	283	CGGCT	3.633	2.941	1.214
18S	288	TTGGT	4.080	2.447	2.811
18S	289	TGGTG	3.294	4.355	1.026
18S	291	GTGAC	2.236	7.393	1.079
18S	298	TAGAT	4.354	4.669	4.277
18S	307	TCGGG	5.403	1.387	2.326
18S	308	CGGGC	4.154	2.594	3.486
18S	309	GGGCC	1.951	1.781	4.167
18S	312	CCGAT	1.800	1.143	2.350
18S	316	TCGCA	1.812	1.575	3.226
18S	320	ACGCC	4.173	2.082	7.292
18S	327	CCGTG	5.103	1.690	4.623
18S	329	GTGGC	1.717	1.222	2.092
18S	330	TGGCG	2.952	1.415	1.301
18S	332	GCGGC	3.510	1.738	1.227
18S	333	CGGCG	5.869	1.807	1.391
18S	335	GCGAC	7.649	1.558	1.871
18S	338	ACGAC	3.303	4.718	4.878
18S	347	TCGAA	2.192	4.751	5.213
18S	351	ACGTC	2.239	0.898	4.000
18S	355	CTGCC	3.096	1.072	6.599
18S	370	TCGAT	2.985	1.945	5.839

18S	373	ATGGT	4.082	0.870	2.203
18S	374	TGGTA	3.809	1.677	1.994
18S	377	TAGTC	2.742	2.008	3.534
18S	380	TCGCC	2.097	1.287	4.194
18S	383	CCGTG	2.492	2.783	4.623
18S	385	GTGCC	2.649	1.980	3.390
18S	394	ATGGT	3.747	1.793	2.203
18S	395	TGGTG	3.596	4.317	1.026
18S	397	GTGAC	3.465	5.056	1.079
18S	403	ACGGG	2.549	2.293	2.791
18S	404	CGGGT	2.812	2.715	2.041
18S	405	GGGTG	2.707	4.381	0.806
18S	407	GTGAC	1.604	3.160	1.079
18S	410	ACGGG	3.100	1.273	2.791
18S	411	CGGGG	2.900	0.638	5.217
18S	412	GGGGA	2.006	1.995	0.769
18S	413	GGGAA	2.086	9.106	2.195
18S	419	CAGGG	3.234	2.352	2.808
18S	420	AGGGT	3.452	1.696	0.000
18S	421	GGGTT	2.122	6.992	2.091
18S	425	TCGAT	2.520	9.170	5.839
18S	431	CCGGA	3.965	1.635	2.721
18S	432	CGGAG	4.193	2.181	4.478
18S	434	GAGAG	2.637	1.424	1.841
18S	436	GAGGG	3.629	1.536	4.667
18S	437	AGGGA	3.794	1.712	1.667
18S	438	GGGAG	3.092	2.320	4.040
18S	440	GAGCC	3.581	2.167	4.918
18S	444	CTGAG	5.150	1.712	2.726
18S	446	GAGAA	5.029	2.937	2.304
18S	451	ACGGC	3.292	2.117	2.500
18S	452	CGGCT	4.030	4.399	1.214
18S	466	AAGGA	2.242	0.506	5.686
18S	467	AGGAA	2.157	0.885	4.787
18S	470	AAGGC	3.015	0.904	2.022
18S	471	AGGCA	2.606	1.834	4.000
18S	474	CAGCA	1.519	1.705	2.936
18S	477	CAGGC	2.939	1.060	3.797
18S	478	AGGCG	3.266	1.811	1.796
18S	480	GCGCG	2.853	1.531	2.881
18S	482	GCGCA	2.155	0.939	2.507
18S	499	CCGAC	2.593	2.939	4.167
18S	504	CCGGG	3.426	0.752	5.769
18S	505	CGGGG	2.602	0.480	5.217
18S	506	GGGGA	2.281	0.752	0.769
18S	507	GGGAG	2.193	0.414	4.040
18S	509	GAGGT	1.920	1.574	2.459
18S	510	AGGTA	1.748	1.351	1.941
18S	513	TAGTG	3.030	1.404	1.437
18S	515	GTGAC	0.797	0.705	1.079
18S	518	ACGAA	1.080	1.278	5.925
18S	534	CAGGA	3.157	6.517	1.282

18S	535	AGGAC	2.307	5.935	2.128
18S	544	TCGAG	2.180	3.362	3.052
18S	546	GAGGC	1.145	4.394	4.038
18S	547	AGGCC	1.538	7.066	5.797
18S	552	CTGTA	3.253	5.362	1.339
18S	558	TTGGA	1.903	1.039	3.889
18S	559	TGGAA	2.072	1.270	2.944
18S	563	ATGAG	1.440	0.848	1.457
18S	565	GAGTC	1.326	0.734	1.766
18S	586	ACGAG	2.391	3.457	3.346
18S	588	GAGGA	1.827	2.878	3.213
18S	589	AGGAT	2.482	2.533	2.935
18S	597	TTGGA	2.917	4.180	3.889
18S	598	TGGAG	2.595	5.579	2.880
18S	600	GAGGG	0.977	2.458	4.667
18S	601	AGGGC	0.946	1.093	0.915
18S	602	GGGCA	1.048	3.116	3.060
18S	606	AAGTC	1.783	1.885	3.763
18S	610	CTGGT	1.704	1.732	0.338
18S	611	TGGTG	1.185	3.529	1.026
18S	613	GTGCC	0.873	3.224	3.390
18S	617	CAGCA	1.512	3.761	2.936
18S	620	CAGCC	1.653	1.160	4.555
18S	623	CCGCG	2.563	0.859	1.667
18S	625	GCGGT	1.839	0.357	1.364
18S	626	CGGTA	0.963	0.596	2.384
18S	635	CAGCT	3.189	1.177	4.023
18S	644	TAGCG	2.944	1.203	1.416
18S	646	GCGTA	2.346	1.947	2.982
18S	656	AAGTT	4.432	1.243	2.556
18S	659	TTGCT	4.311	5.902	3.071
18S	662	CTGCA	3.110	9.356	1.826
18S	665	CAGTT	2.067	4.259	1.926
18S	673	AAGCT	2.177	4.550	3.448
18S	677	TCGTA	2.735	12.441	2.104
18S	680	TAGTT	4.086	5.772	1.348
18S	683	TTGGA	1.425	3.857	3.889
18S	684	TGGAT	1.533	4.394	1.790
18S	690	TTGGG	3.573	3.859	0.935
18S	691	TGGGA	3.097	4.348	1.542
18S	692	GGGAG	2.840	4.130	4.040
18S	694	GAGCG	3.597	1.844	4.595
18S	696	GCGGG	3.496	1.655	5.556
18S	697	CGGGC	2.967	0.989	3.486
18S	698	GGGCG	1.864	0.675	2.020
18S	700	GCGGG	1.939	1.134	5.556
18S	701	CGGGC	2.775	1.186	3.486
18S	702	GGGCG	3.124	1.955	2.020
18S	704	GCGGT	2.160	2.740	1.364
18S	705	CGGTC	2.834	4.725	5.469
18S	709	CCGCC	3.536	4.306	8.589
18S	712	CCGCG	4.408	5.183	1.667

18S	714	GCGAG	2.374	6.317	3.970
18S	716	GAGGC	4.466	4.226	4.038
18S	717	AGGCG	4.027	3.538	1.796
18S	719	GCGAG	4.932	6.427	3.970
18S	721	GAGCC	6.241	7.016	4.918
18S	727	CCGCC	5.246	1.634	8.589
18S	731	CCGTC	2.333	1.121	3.694
18S	737	CCGCC	2.158	1.399	8.589
18S	744	TTGCC	2.127	2.287	2.743
18S	751	TCGGC	4.616	1.049	2.504
18S	752	CGGCG	3.006	3.263	1.391
18S	754	GCGCC	2.532	1.174	3.614
18S	762	TCGAT	3.563	2.092	5.839
18S	765	ATGCT	6.212	4.223	4.854
18S	772	TAGCT	4.541	2.945	4.770
18S	775	CTGAG	3.378	3.672	2.726
18S	777	GAGTG	3.558	3.145	2.174
18S	779	GTGTC	3.436	4.656	1.511
18S	784	CCGCG	4.655	2.041	1.667
18S	786	GCGGG	2.133	0.319	5.556
18S	787	CGGGG	1.493	1.163	5.217
18S	788	GGGGC	0.974	1.681	2.646
18S	789	GGGCC	0.731	1.569	4.167
18S	793	CCGAA	2.047	2.149	4.430
18S	796	AAGCG	2.356	0.910	0.974
18S	798	GCGTT	4.297	0.927	1.471
18S	807	TTGAA	3.100	5.529	5.033
18S	817	TAGAG	1.401	4.496	0.606
18S	819	GAGTG	3.506	7.856	2.174
18S	821	GTGTT	2.896	6.080	1.323
18S	828	AAGCA	2.685	2.760	3.790
18S	831	CAGGC	2.337	3.073	3.797
18S	832	AGGCC	2.802	4.247	5.797
18S	836	CCGAG	2.911	5.043	6.192
18S	838	GAGCC	3.721	2.480	4.918
18S	841	CCGCC	4.777	4.610	8.589
18S	845	CTGGA	3.414	2.029	1.197
18S	846	TGGAT	2.744	2.693	1.790
18S	852	CCGCA	3.714	3.779	2.484
18S	855	CAGCT	2.857	2.109	4.023
18S	859	TAGGA	3.491	0.708	4.368
18S	860	AGGAA	3.556	0.775	4.787
18S	867	ATGGA	1.876	0.962	2.826
18S	868	TGGAA	2.366	3.538	2.944
18S	873	TAGGA	3.158	1.645	4.368
18S	874	AGGAC	2.652	2.194	2.128
18S	878	CCGCG	1.211	0.687	1.667
18S	880	GCGGT	1.548	1.028	1.364
18S	881	CGGTT	1.328	2.429	2.116
18S	891	TTGTT	6.349	2.567	1.619
18S	894	TTGGT	3.001	3.590	2.811
18S	895	TGGTT	1.906	7.224	1.856

18S	901	TCGGA	2.294	1.100	2.703
18S	902	CGGAA	1.734	1.913	1.050
18S	907	CTGAG	3.119	0.669	2.726
18S	909	GAGGC	2.309	1.647	4.038
18S	910	AGGCC	2.127	2.609	5.797
18S	915	ATGAT	2.002	4.084	5.227
18S	921	AAGAG	3.816	4.153	1.693
18S	923	GAGGG	3.164	2.354	4.667
18S	924	AGGGA	3.504	2.544	1.667
18S	925	GGGAC	2.781	4.158	1.575
18S	928	ACGGC	3.753	6.054	2.500
18S	929	CGGCC	2.248	5.178	0.813
18S	932	CCGGG	2.102	0.893	5.769
18S	933	CGGGG	1.871	0.998	5.217
18S	934	GGGGG	1.259	1.870	6.000
18S	935	GGGGC	0.803	4.541	2.646
18S	936	GGGCA	0.728	9.199	3.060
18S	942	TCGTA	3.587	2.610	2.104
18S	947	TTGCG	4.909	2.849	1.813
18S	949	GCGCC	3.415	2.479	3.614
18S	952	CCGCT	3.651	2.484	3.141
18S	956	TAGAG	3.539	1.995	0.606
18S	958	GAGGT	1.772	1.014	2.459
18S	959	AGGTG	1.762	2.246	0.951
18S	961	GTGAA	1.772	3.836	3.093
18S	970	TTGGA	6.425	10.886	3.889
18S	971	TGGAC	5.374	12.426	1.958
18S	975	CCGGC	2.438	4.062	2.588
18S	976	CGGCG	3.102	4.118	1.391
18S	978	GCGCA	9.967	4.538	2.507
18S	982	AAGAC	2.382	3.259	2.307
18S	985	ACGGA	3.058	4.035	3.125
18S	986	CGGAC	2.008	3.257	1.775
18S	991	CAGAG	1.600	0.941	1.225
18S	993	GAGCG	2.950	0.297	4.595
18S	995	GCGAA	2.962	0.618	2.219
18S	999	AAGCA	2.602	0.591	3.790
18S	1005	TTGCC	3.279	0.554	2.743
18S	1010	AAGAA	1.163	0.542	4.916
18S	1014	ATGTT	3.636	0.611	2.422
18S	1029	AAGAA	1.404	3.238	4.916
18S	1033	ACGAA	5.622	1.592	5.925
18S	1037	AAGTC	2.539	3.485	3.763
18S	1040	TCGGA	2.775	2.036	2.703
18S	1041	CGGAG	2.771	2.120	4.478
18S	1043	GAGGT	1.624	1.306	2.459
18S	1044	AGGTT	2.260	4.256	2.881
18S	1048	TCGAA	1.966	2.156	5.213
18S	1051	AAGAC	1.841	0.883	2.307
18S	1054	ACGAT	2.956	0.553	6.569
18S	1059	CAGAT	2.827	0.547	2.605
18S	1065	CCGTC	3.330	0.211	3.694

18S	1068	TCGTA	2.370	0.453	2.104
18S	1071	TAGTT	2.814	0.867	1.348
18S	1076	CCGAC	3.050	3.066	4.167
18S	1086	ACGAT	3.852	2.359	6.569
18S	1089	ATGCC	3.803	3.428	8.696
18S	1092	CCGAC	2.380	3.033	4.167
18S	1096	CCGGC	4.633	10.871	2.588
18S	1097	CGGCG	5.026	10.913	1.391
18S	1099	GCGAT	1.358	1.939	2.802
18S	1102	ATGCG	3.222	0.904	1.871
18S	1104	GCGGC	3.269	0.395	1.227
18S	1105	CGGCG	4.887	0.398	1.391
18S	1107	GCGGC	4.117	0.545	1.227
18S	1108	CGGCG	3.154	1.027	1.391
18S	1110	GCGTT	4.216	2.655	1.471
18S	1121	ATGAC	2.471	7.448	3.482
18S	1126	CCGCC	3.158	1.707	8.589
18S	1129	CCGGG	2.718	2.391	5.769
18S	1130	CGGGC	2.391	2.971	3.486
18S	1131	GGGCA	2.862	5.291	3.060
18S	1134	CAGCT	3.671	1.836	4.023
18S	1140	CCGGG	4.555	0.644	5.769
18S	1141	CGGGA	3.529	0.997	2.429
18S	1142	GGGAA	2.634	1.585	2.195
18S	1151	AAGTC	1.793	0.940	3.763
18S	1157	TTGGG	5.805	1.820	0.935
18S	1158	TGGGT	2.224	2.576	1.977
18S	1159	GGGTT	1.946	3.423	2.091
18S	1164	CCGGG	3.024	0.884	5.769
18S	1165	CGGGG	2.783	0.404	5.217
18S	1166	GGGGG	1.880	0.405	6.000
18S	1167	GGGGG	0.611	0.403	6.000
18S	1168	GGGGA	0.536	0.646	0.769
18S	1169	GGGAG	0.895	1.128	4.040
18S	1171	GAGTA	1.091	2.126	2.726
18S	1175	ATGGT	2.424	1.254	2.203
18S	1176	TGGTT	1.772	1.370	1.856
18S	1179	TTGCA	1.684	0.887	3.339
18S	1184	AAGCT	4.375	2.350	3.448
18S	1187	CTGAA	1.996	3.933	2.784
18S	1197	AAGGA	10.488	5.470	5.686
18S	1198	AGGAA	9.849	8.096	4.787
18S	1203	TTGAC	2.577	7.759	3.569
18S	1206	ACGGA	2.542	0.878	3.125
18S	1207	CGGAA	2.351	0.449	1.050
18S	1210	AAGGG	2.684	0.283	1.026
18S	1211	AGGGC	3.317	0.472	0.915
18S	1212	GGGCA	3.354	0.755	3.060
18S	1221	CAGGA	4.687	0.793	1.282
18S	1222	AGGAG	4.318	1.144	3.512
18S	1224	GAGTG	4.021	0.213	2.174
18S	1226	GTGGA	5.112	0.228	3.209

18S	1227	TGGAG	4.787	0.166	2.880
18S	1229	GAGCC	2.888	0.352	4.918
18S	1233	CTGCG	2.099	0.373	0.524
18S	1235	GCGGC	2.232	0.275	1.227
18S	1236	CGGCT	2.156	0.442	1.214
18S	1245	TTGAC	2.914	1.224	3.569
18S	1255	ACGGG	4.253	2.086	2.791
18S	1256	CGGGA	2.198	1.498	2.429
18S	1257	GGGAA	2.449	3.434	2.195
18S	1269	CCGGC	3.041	2.623	2.588
18S	1270	CGGCC	3.491	2.207	0.813
18S	1274	CCGGA	1.712	4.774	2.721
18S	1275	CGGAC	1.639	4.743	1.775
18S	1280	ACGGA	3.440	1.731	3.125
18S	1281	CGGAC	2.105	1.828	1.775
18S	1285	CAGGA	3.304	1.005	1.282
18S	1286	AGGAT	2.623	0.534	2.935
18S	1290	TTGAC	5.311	1.842	3.569
18S	1294	CAGAT	2.459	1.140	2.605
18S	1298	TTGAT	3.452	1.115	2.577
18S	1302	TAGCT	1.503	1.543	4.770
18S	1312	TCGAT	2.419	2.839	5.839
18S	1318	CCGTG	2.773	2.765	4.623
18S	1320	GTGGG	1.891	2.670	0.000
18S	1321	TGGGT	1.825	2.502	1.977
18S	1322	GGGTG	1.410	1.948	0.806
18S	1324	GTGGT	1.041	0.697	1.974
18S	1325	TGGTG	1.439	1.487	1.026
18S	1327	GTGGT	1.024	2.081	1.974
18S	1328	TGGTG	0.942	2.291	1.026
18S	1330	GTGCA	0.592	2.837	2.222
18S	1334	ATGGC	1.472	4.113	2.869
18S	1335	TGGCC	1.261	2.155	2.827
18S	1338	CCGTT	1.168	4.683	2.993
18S	1345	TAGTT	3.759	7.136	1.348
18S	1348	TTGGT	4.255	3.912	2.811
18S	1349	TGGTG	3.564	3.255	1.026
18S	1351	GTGGA	2.276	2.913	3.209
18S	1352	TGGAG	2.792	4.499	2.880
18S	1354	GAGCG	1.513	3.480	4.595
18S	1356	GCGAT	1.663	3.033	2.802
18S	1361	TTGTC	3.182	1.623	2.079
18S	1365	CTGGT	2.430	0.950	0.338
18S	1366	TGGTT	3.359	2.516	1.856
18S	1375	CCGAT	2.953	6.210	2.350
18S	1381	ACGAA	3.899	4.902	5.925
18S	1385	ACGAG	1.298	0.688	3.346
18S	1387	GAGAC	1.772	0.990	1.538
18S	1393	CTGGC	1.832	0.598	2.083
18S	1394	TGGCA	2.533	0.441	3.233
18S	1398	ATGCT	2.604	0.913	4.854
18S	1406	TAGTT	5.060	2.923	1.348

18S	1411	ACGCG	4.124	3.834	4.640
18S	1413	GCGAC	5.335	6.462	1.871
18S	1420	CCGAG	2.603	15.587	6.192
18S	1422	GAGCG	2.437	6.717	4.595
18S	1424	GCGGT	1.394	2.541	1.364
18S	1425	CGGTC	3.157	1.958	5.469
18S	1428	TCGGC	2.886	1.332	2.504
18S	1429	CGGCG	1.795	0.814	1.391
18S	1431	GCGTC	2.258	1.466	1.455
18S	1447	TAGAG	3.206	2.071	0.606
18S	1449	GAGGG	1.883	1.786	4.667
18S	1450	AGGGA	3.822	3.753	1.667
18S	1451	GGGAC	2.784	3.678	1.575
18S	1456	AAGTG	3.349	4.024	1.102
18S	1458	GTGGC	0.949	4.394	2.092
18S	1459	TGGCG	0.847	5.038	1.301
18S	1461	GCGTT	0.575	5.204	1.471
18S	1466	CAGCC	1.532	2.913	4.555
18S	1473	CCGAG	1.746	1.721	6.192
18S	1475	GAGAT	1.317	2.023	2.734
18S	1479	TTGAG	3.089	2.115	1.671
18S	1481	GAGCA	2.946	2.223	3.951
18S	1490	CAGGT	4.417	0.811	3.125
18S	1491	AGGTC	4.896	1.043	6.490
18S	1495	CTGTG	3.965	1.557	1.541
18S	1497	GTGAT	1.494	1.606	2.367
18S	1500	ATGCC	2.038	2.043	8.696
18S	1507	TAGAT	1.055	5.377	4.277
18S	1510	ATGTC	3.011	3.574	5.270
18S	1514	CCGGG	2.234	0.845	5.769
18S	1515	CGGGG	1.638	1.055	5.217
18S	1516	GGGGC	1.752	1.421	2.646
18S	1517	GGGCT	1.662	2.120	1.845
18S	1520	CTGCA	1.822	2.454	1.826
18S	1524	ACGCG	1.904	2.932	4.640
18S	1526	GCGCG	2.936	3.189	2.881
18S	1528	GCGCT	3.273	2.337	2.963
18S	1536	CTGAC	3.307	2.777	2.013
18S	1540	CTGGC	3.482	1.692	2.083
18S	1541	TGGCT	3.696	2.771	1.397
18S	1546	CAGCG	3.363	1.115	1.457
18S	1548	GCGTG	2.995	0.970	2.277
18S	1550	GTGTG	2.479	1.898	3.030
18S	1552	GTGCC	2.257	2.196	3.390
18S	1563	ACGCC	1.892	3.354	7.292
18S	1566	CCGGC	1.877	2.419	2.588
18S	1567	CGGCA	4.031	4.551	2.367
18S	1570	CAGGC	1.558	1.701	3.797
18S	1571	AGGCG	2.146	1.542	1.796
18S	1573	GCGCG	2.345	1.400	2.881
18S	1575	GCGGG	4.710	1.038	5.556
18S	1576	CGGGT	3.463	1.586	2.041

18S	1577	GGGTA	4.433	2.513	1.050
18S	1584	CCGTT	4.027	0.764	2.993
18S	1587	TTGAA	3.218	2.898	5.033
18S	1598	TCGTG	4.248	10.159	3.325
18S	1600	GTGAT	3.013	7.938	2.367
18S	1603	ATGGG	2.637	2.362	2.190
18S	1604	TGGGG	2.970	1.886	0.000
18S	1605	GGGGA	2.559	1.729	0.769
18S	1606	GGGAT	1.511	1.791	1.140
18S	1610	TCGGG	2.209	0.443	2.326
18S	1611	CGGGG	2.614	0.889	5.217
18S	1612	GGGGA	1.029	0.669	0.769
18S	1613	GGGAT	0.780	0.696	1.140
18S	1617	TTGCA	0.541	1.023	3.339
18S	1632	ATGAA	1.119	3.890	5.233
18S	1636	ACGAG	1.238	1.484	3.346
18S	1638	GAGGA	4.894	1.347	3.213
18S	**1639**	**AGGAA**	**61.300**	**3.219**	**4.787**
18S	1648	CAGTA	1.775	3.550	4.176
18S	1652	AAGTG	3.788	2.426	1.102
18S	1654	GTGCG	2.216	1.460	2.076
18S	1656	GCGGG	1.594	1.592	5.556
18S	1657	CGGGT	1.687	2.150	2.041
18S	1658	GGGTC	1.546	1.864	2.791
18S	1665	AAGCT	3.155	1.743	3.448
18S	1669	TTGCG	3.256	1.722	1.813
18S	1671	GCGTT	3.486	1.236	1.471
18S	1674	TTGAT	3.453	2.268	2.577
18S	1680	AAGTC	0.715	2.062	3.763
18S	1686	CTGCC	1.484	5.946	6.599
18S	1693	TTGTA	5.278	22.013	2.550
18S	1702	CCGCC	4.434	4.870	8.589
18S	1706	CCGTC	3.571	0.822	3.694
18S	1709	TCGCT	3.553	1.322	9.829
18S	1718	CCGAT	4.117	5.189	2.350
18S	1722	TTGGA	3.166	3.539	3.889
18S	1723	TGGAT	3.469	2.984	1.790
18S	1726	ATGGT	2.884	2.999	2.203
18S	1727	TGGTT	3.037	5.124	1.856
18S	1732	TAGTG	5.646	3.072	1.437
18S	1734	GTGAG	3.042	2.372	2.821
18S	1736	GAGGC	1.469	7.174	4.038
18S	1737	AGGCC	2.454	10.509	5.797
18S	1743	TCGGA	4.890	3.154	2.703
18S	1744	CGGAT	3.114	1.815	2.100
18S	1748	TCGGC	3.609	3.919	2.504
18S	1749	CGGCC	2.210	3.247	0.813
18S	1754	CCGCC	6.055	3.221	8.589
18S	1757	CCGGG	2.329	2.868	5.769
18S	1758	CGGGG	2.894	3.202	5.217
18S	1759	GGGGT	1.958	2.413	0.000
18S	1760	GGGTC	2.464	2.085	2.791

18S	1763	TCGGC	4.840	4.358	2.504
18S	1764	CGGCC	3.501	4.655	0.813
18S	1770	ACGGC	5.024	3.081	2.500
18S	1771	CGGCC	3.932	3.588	0.813
18S	1776	CTGGC	3.401	3.433	2.083
18S	1777	TGGCG	4.809	2.891	1.301
18S	1779	GCGGA	2.842	1.804	1.389
18S	1780	CGGAG	3.234	2.636	4.478
18S	1782	GAGCG	1.285	2.959	4.595
18S	1784	GCGCT	2.239	2.413	2.963
18S	1787	CTGAG	1.723	2.404	2.726
18S	1789	GAGAA	1.453	2.867	2.304
18S	1792	AAGAC	3.277	1.061	2.307
18S	1795	ACGGT	3.558	1.114	3.200
18S	1796	CGGTC	2.638	0.852	5.469
18S	1799	TCGAA	2.370	2.281	5.213
18S	1805	TTGAC	4.762	3.614	3.569
18S	1814	TAGAG	2.549	1.746	0.606
18S	1816	GAGGA	2.705	1.126	3.213
18S	1817	AGGAA	2.373	1.168	4.787
18S	1820	AAGTA	1.096	1.197	5.179
18S	1826	AAGTC	3.369	3.938	3.763
18S	1829	TCGTA	3.003	4.807	2.104
18S	1836	AAGGT	1.251	0.341	2.528
18S	1837	AGGTT	1.061	0.448	2.881
18S	1843	CCGTA	1.359	3.798	7.973
18S	1846	TAGGT	3.874	1.067	2.344
18S	1847	AGGTG	4.589	0.992	0.951
18S	1849	GTGAA	1.487	1.356	3.093
18S	1855	CTGCG	0.154	0.061	0.524
18S	1857	GCGGA	0.044	0.321	1.389
18S	1858	CGGAA	0.178	0.227	1.050
18S	1861	AAGGA	0.210	0.034	5.686
18S	1862	AGGAT	0.166	0.176	2.935
